# Enhanced Cross-Presentation and Improved CD8^+^ T Cell Responses after Mannosylation of Synthetic Long Peptides in Mice

**DOI:** 10.1371/journal.pone.0103755

**Published:** 2014-08-19

**Authors:** Judith Rauen, Christoph Kreer, Arlette Paillard, Suzanne van Duikeren, Willemien E. Benckhuijsen, Marcel G. Camps, A. Rob P. M. Valentijn, Ferry Ossendorp, Jan W. Drijfhout, Ramon Arens, Sven Burgdorf

**Affiliations:** 1 Life and Medical Sciences Institute, University of Bonn, Bonn, Germany; 2 Department of Immunohematology and Blood Transfusion, Leiden University Medical Center, Leiden, the Netherlands; 3 Department of Bio-organic Synthesis, Leiden University Medical Center, Leiden, the Netherlands; MRC National Institute for Medical Research, United Kingdom

## Abstract

The use of synthetic long peptides (SLP) has been proven to be a promising approach to induce adaptive immune responses in vaccination strategies. Here, we analyzed whether the efficiency to activate cytotoxic T cells by SLP-based vaccinations can be increased by conjugating SLPs to mannose residues. We could demonstrate that mannosylation of SLPs results in increased internalization by the mannose receptor (MR) on murine antigen-presenting cells. MR-mediated internalization targeted the mannosylated SLPs into early endosomes, from where they were cross-presented very efficiently compared to non-mannosylated SLPs. The influence of SLP mannosylation was specific for cross-presentation, as no influence on MHC II-restricted presentation was observed. Additionally, we showed that vaccination of mice with mannosylated SLPs containing epitopes from either ovalbumin or HPV E7 resulted in enhanced proliferation and activation of antigen-specific CD8^+^ T cells. These findings demonstrate that mannosylation of SLPs augments the induction of a cytotoxic T cell response *in vitro* and *in vivo* and might be a promising approach to induce cytotoxic T cell responses in e.g. cancer therapy and anti-viral immunity.

## Introduction

Both prophylactic and therapeutic vaccinations are successful approaches to induce humoral and cellular adaptive immune responses aiming to control infectious pathogens and cancerous cells [1]. Depending on the nature of the pathogen or cancer type, antigen-specific B cell, CD4^+^ and/or CD8^+^ T cell responses are critical to confer protective immunity. The induction of proper humoral immunity by prophylactic vaccines has shown its value greatly, as it resulted in eradication or at least strong reduction of a number of devastating pathogen-caused diseases including diphtheria, measles, mumps, pertussis, polio, rubella and small pox [1]. Immunity against certain (persistent) intracellular bacteria and viruses as well as most cancers, however, relies highly on the activation of antigen-specific CD4^+^ and CD8^+^ T cells, which requires different types of vaccines [2]. To this end, diverse (therapeutic) vaccine methodologies have been developed with varying success that aim to mount a robust and effective T cell response with the ultimate aim to prevent and/or treat infections and cancers.

Vaccination with synthetic long peptides (SLPs), covering viral or tumor epitopes, has shown promising results in experimental models and recently also in clinical therapeutic vaccination trials [3]. This peptide-based vaccine platform allows covering multiple (overlapping) MHC classes I and II epitopes and therefore does not require the necessity for HLA-typing for each patient to be treated [4], [5]. Moreover, in contrast to short peptides, SLPs are not able to bind directly to MHC class I and their presentation to CD8^+^ T cells therefore requires uptake and processing by antigen-presenting cells (APCs) such as dendritic cells (DCs) before they are presented. This is advantageous, as properly activated dendritic cells (DCs) are vital for the strength of the ensuing T-cell responses [6]. Other immune cells for example generally lack the capacity to provide adequate T cell costimulation and thus may cause tolerance. The efficacy of a particular peptide vaccine is however influenced by many parameters and its success ranges from inducing clinical efficacy to detrimental effects such as hyperreactivity and hyporesponsiveness [7], [8], [9], [10], [11]. Mechanistic studies with peptide vaccines in different experimental models revealed that by more specific targeting, improving the uptake of SLPs, and/or activation of APCs the SLP-based vaccines generally advance leading to better clinical success [5].

An effective manner of improving SLP-based vaccines is the addition or conjugation of adjuvants [12], [13]. Especially, adjuvants that activate APCs by triggering the Toll-like receptors 3 and 9, such as poly∶IC, and CpG, respectively, which are expressed by APCs, significantly improve SLP effectiveness [14]. A different approach is the targeting of the internalization system of DCs. In this respect the mannose receptor (MR), an endocytic receptor expressed by dendritic cells that is involved in binding and uptake of carbohydrates and related molecules [15], is of great interest.

The MR is a member of the C-type lectin family consisting of an N-terminal cystein-rich domain, a fibronectin type II repeat domain, eight carbohydrate recognition domains (CRD), a transmembrane domain and a short intracellular region [16]. Through CRD4, the MR binds glycosylated proteins terminated in mannose, fucose or GlcNAc, which leads to their internalisation. In previous studies, we could demonstrate a close correlation between the MR and antigen cross-presentation. Whereas antigens internalised by fluid phase pinocytosis or by scavenger receptors are targeted rapidly towards lysosomes for degradation and subsequent presentation on MHC II molecules, antigens internalized by the MR are directed towards a separate subset of early endosomes, where they are rescued from lysosomal degradation and from where they are processed for cross-presentation on MHC I [17], [18], [19]. This connection between the MR and cross-presentation made the MR a promising subject for antigen targeting studies in approaches aimed at the induction of a strong cytotoxic T cell response, like in anti-tumour therapies [20].

In this study, we tried to improve SLP-based vaccination strategies by targeting SLPs towards the MR by conjugation to bis-mannose. We demonstrated both *in vitro* and *in vivo* that such mannosylation leads to increased cross-presentation of the SLPs and enhanced SLP-specific CD8^+^ T cell activation and hence might be an efficient way to improve SLP-based vaccination strategies.

## Materials and Methods

### Mice

C57BL/6, MR^−/−^, OT-I and OT-II mice were maintained in the central animal facility of the LIMES Institute or the Leiden University Medical Center (LUMC). All mice were housed in specific pathogen-free conditions and used at 8–10 weeks of age. All animal experiments were approved by the Animal Experiments Committee of the LUMC and performed according to the guide to animal experimentation set by the LUMC and to the Dutch Experiments on Animals Act that serves the implementation of ‘Guidelines on the protection of experimental animals’ by the Council of Europe.

### Generation of BM-DCs

BM-DCs were generated using a GM-CSF-producing cell line as described before [15].

### Synthesis of mannosylated and non-mannosylated peptides

Peptides were prepared using solid-phase synthesis. Mannosylation was accomplished by N-terminal elongation of the peptide with a building block containing lysine coupled to two tetraacetyl-protected mannose groups. The acetyl protecting groups on the mannose moieties were removed using Tesser's base. Peptides were analyzed using UPLC-MS and Maldi-Tof mass spectrometry. Alexa647 modified peptides were prepared from the corresponding cysteine-containing precursor peptides. The cysteine-containing peptides were treated overnight with an excess of AlexaFluor647 C2-maleimide (A20347, Molecular Probes) in a mixture of DMSO and a sodiumphosphate buffer at pH 7.6–8.3. The Alexa647 labeled peptides obtained were subsequently purified using reversed phase chromatography using a C18 column. The structure of the labeled peptides was verified using Maldi-Tof mass spectrometry. Purity of the generated peptides was >80%.

### Flow cytometric analysis of peptide uptake

BM-DCs were incubated with 250 nM Alexa Fluor 647-labeled SLPs or 250 ng/ml Alexa Fluor 647-labeled OVA for 15 min at 37°C. Cells were harvested, washed and antigen uptake monitored by flow cytometry. Mean fluorescence intensity was calculated using FlowJo software.

### Intracellular distribution of SLPs by immunofluorescence microscopy

Cells were pulsed for 15 min with 200 nM fluorochrome-labeled SLPs and chased for another 20 min with medium. Staining experiments were performed as described before [17]. For co-localizing experiments with Transferrin, OVA or Lucifer Yellow (LY), fluorochrome-labeled SLPs were incubated together with 5 µg/ml fluorochrome-labeled Transferrin, 250 ng/ml fluorochrome-labeled OVA or 0,3 mg/ml lucifer yellow. Nuclei were visualized with 1 µg/ml of the DNA-intercalating dye 4,6-diamidino-2-phenylindole (DAPI). Cells were analyzed with an ApoTome microscope (Zeiss). Co-localization was quantified by calculating the Pearson correlation coefficient and Mander's overlap coefficient using the ImageJ software.

### Proliferation of antigen-specific T cells

BM-DCs from wildtype or MR-deficient mice were incubated with 200 nM mannosylated or non-mannosylated SLPs or 0,5 mg/ml OVA for 2 h and co-cultivated with CFSE-labeled OT-I or OT-II T cells (labeled 15 min with 2 µM CFSE) for 3 days. Proliferation was assessed by flow cytometric analysis of the CFSE dilution. Division index and percentage dividing cells were calculated using the FlowJo software.

### Peptide immunization and detection of in vivo T cell expansion

Mannosylated or non-mannosylated SLPs were dissolved in PBS. Per mouse, 75 µg SLPs were injected subcutaneously (s.c.) or intradermally (i.d.) in a total volume of 200 µl or 50 µl, respectively, with CpG as adjuvant (ODN1826, type B; 20 µg per mouse; purchased from InvivoGen). At day 7 post-vaccination, cell surface staining was performed on freshly prepared peripheral blood mononuclear cells and splenocytes after red blood cell lysis. Cells were surface stained for 30 minutes with allophycocyanin-labeled OVA_257–264_ or E7_49–57_ tetramers and fluorescently labeled antibodies specific for mouse CD3 and CD8 in staining buffer (PBS containing 1% FCS and 0.05% sodium azide). 7-AAD (Life Technologies) was used for dead cell exclusion. Flow cytometric intracellular cytokine analysis of peripheral blood mononuclear cells and splenocytes was performed after 5 h stimulation with the short HPV16 E7_49–57_ peptide (5 µg/ml) in presence of Brefeldin A (2 µg/ml). After cell surface staining with fluorescently labeled antibodies to mouse CD8, cells were fixed with Cytofix/Cytoperm solution (BD Biosciences), and permeabilized with Perm/Wash buffer. Subsequently, cells were stained for 30 min at 4°C with fluorescently labeled antibodies against IFN-γ and TNFα. Samples were acquired with a BD LSRII Flowcytometer and results were analyzed using FlowJo software (Treestar).

### Antibodies and MHC class I tetramers

Rat anti-MR (MR5D3) and rat anti-CD86 (PO3) were from AbD Serotec; rat anti-LAMP-1 (1D4B) from BD; rabbit anti-EEA1 (H-300) from Santa Cruz; rabbit anti-LY (A5751) from Life Technologies; rat anti-CD8 (53-6.7), rat anti-CD40 (1C10), mouse anti-MHC I (28-14-8) and hamster anti-CD80 (16-10A1) were from eBiosciences and all secondary antibodies from Life Technologies. H-2K^b^ OVA_257–264_ and H-2D^b^ E7_49–57_ tetramers were generated according to standard procedures. Fluorescently labeled antibodies against mouse CD3, CD8, IFN-γ and TNFα were purchased from eBioscience and BD Pharmingen

### Statistical analysis

P values were calculated by two-tailed T test (n≥3) using Excel (Microsoft) or Prism (Graphpad) Software.

## Results

### Increased MR-mediated uptake of mannosylated SLPs

**Figure 1:**
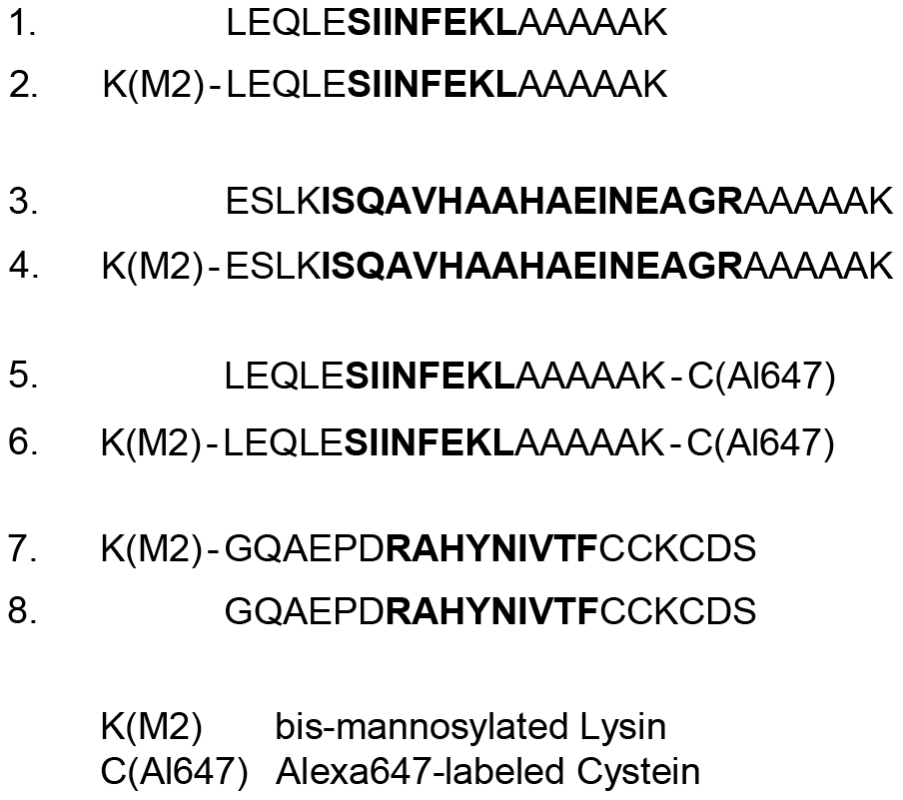
Overview of the used SLPs. To study the effect of mannosylation on cross-presentation, we generated mannosylated and non-mannosylated SLPs containing the MHC I-restricted epitope of OVA (OVA_257–264_; peptides 1 and 2). Mannosylation was introduced by a bis-mannosylated Lysin residue. Additionally, we generated mannosylated or non-mannosylated SLPs containing the MHC II-restricted epitope of OVA (OVA_323–339_; peptides 3 and 4). To analyze intracellular trafficking, we synthesized fluorescently-labeled mannosylated or non-mannosylated SLPs (peptides 5 and 6). The Alexa647 fluorochrome was introduced by conjugation to an Alexa647-labeled cysteine. To study T cell responses against the E7 protein of HPV16, we generated mannosylated or non-mannosylated SLPs containing the MHC I restricted epitope of E7 (E7_43–63_; peptides 7 and 8). Epitopes within the SLPs are in bold.

To investigate whether mannosylation of SLPs influenced their presentation and the ensuing T cell activation, we synthesized different mannosylated and non-mannosylated peptides (Fig. 1). We generated mannosylated and non-mannosylated peptides containing the MHC I-restricted epitope (peptides 1 and 2 in Fig. 1) or the MHC II-restricted epitope (peptides 3 and 4 in Fig. 1) of OVA. To monitor antigen uptake and routing, we additionally conjugated mannosylated or non-mannosylated peptides to the fluorochrome Alexa647 (peptides 5 and 6 in Fig. 1). Finally, to investigate T cell responses against antigens other than OVA, we generated mannosylated or non-mannosylated SLPs containing the MHC I-restricted epitope of the E7 protein of the human papilloma virus (HPV) (peptides 7 and 8).

**Figure 2:**
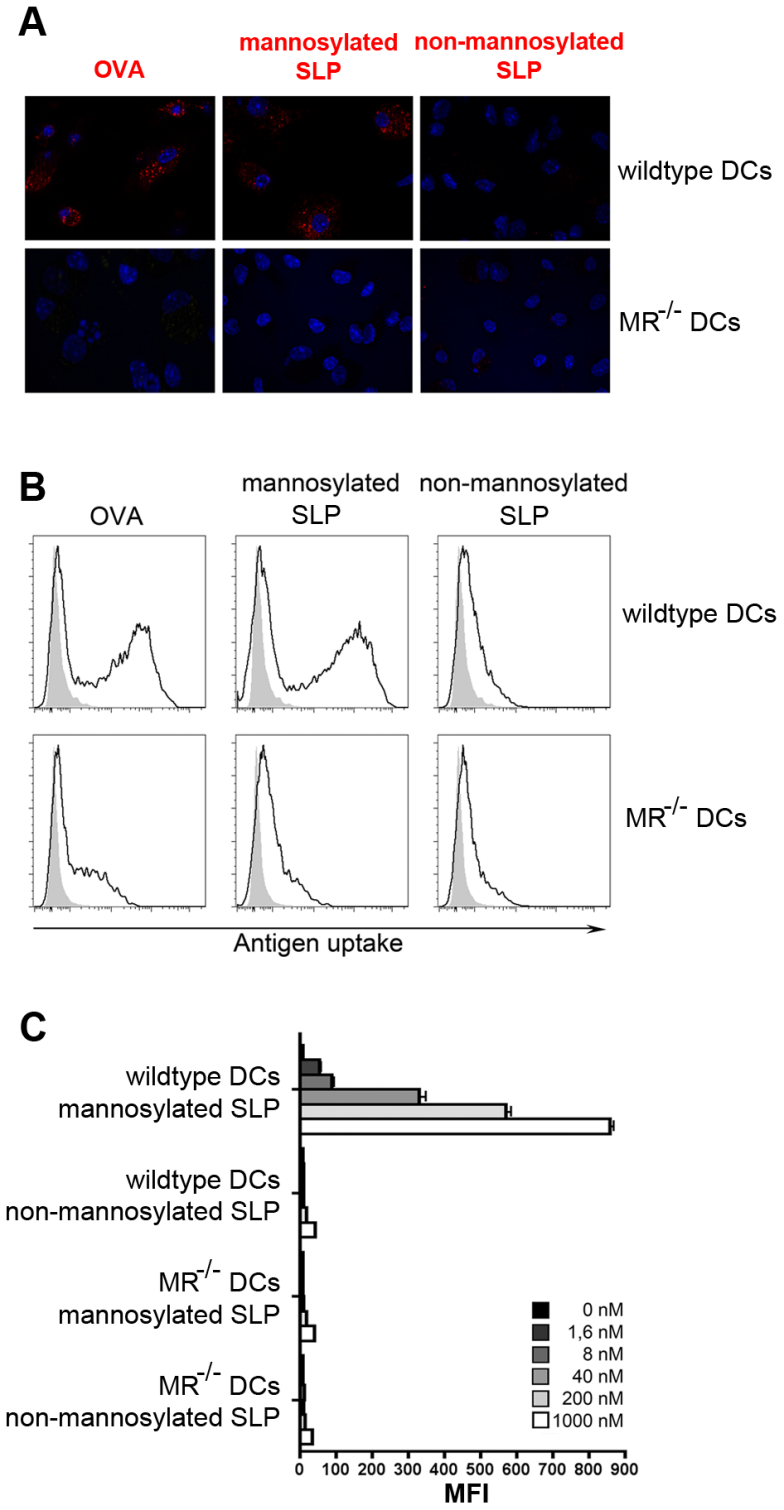
Uptake of mannosylated and non-mannosylated SLPs. A) Wildtype or MR-deficient BM-DCs were incubated with 250 ng/ml fluorochrome-labeled OVA, 200 nM mannosylated SLPs or non-mannosylated SLPs for 15 min, chased with medium for 20 min and analyzed by immunofluorescence microscopy. Nuclei stained with DAPI are depicted in blue. B) Wildtype or MR-deficient BM-DCs were incubated with 250 ng/ml fluorochrome-labeled OVA, 200 nM mannosylated SLPs or non-mannosylated SLPs for 15 min. Antigen uptake was monitored by flow cytometry (gated on all living cells). C) Quantification of B) using different antigen concentrations. Depicted are representative results of at least 3 independent experiments. MFI: mean fluorescence intensity.

First, we analyzed whether mannosylation resulted in altered endocytic properties of the SLPs. To this end, we incubated bone marrow-derived DCs (BM-DCs) with fluorochrome-labeled SLPs and analyzed uptake by immunofluorescence microscopy (Fig. 2A) and flow cytometry (Fig. 2B, 2C, S1). Whereas no significant uptake of non-mannosylated peptides could be observed, we detected a clear internalization of mannosylated SLPs. This uptake was comparable to the internalization of OVA, which is highly mannosylated in its natural form [21], indicating that mannosylation of SLPs results in enhanced uptake. The specific increase in uptake of mannosylated peptides was not observed using BM-DCs from MR-deficient mice (Fig. 2A, 2B, 2C, S1), demonstrating that enhanced uptake was due to MR-mediated endocytosis.

### Mannosylation targets SLPs towards early endosomes

**Figure 3:**
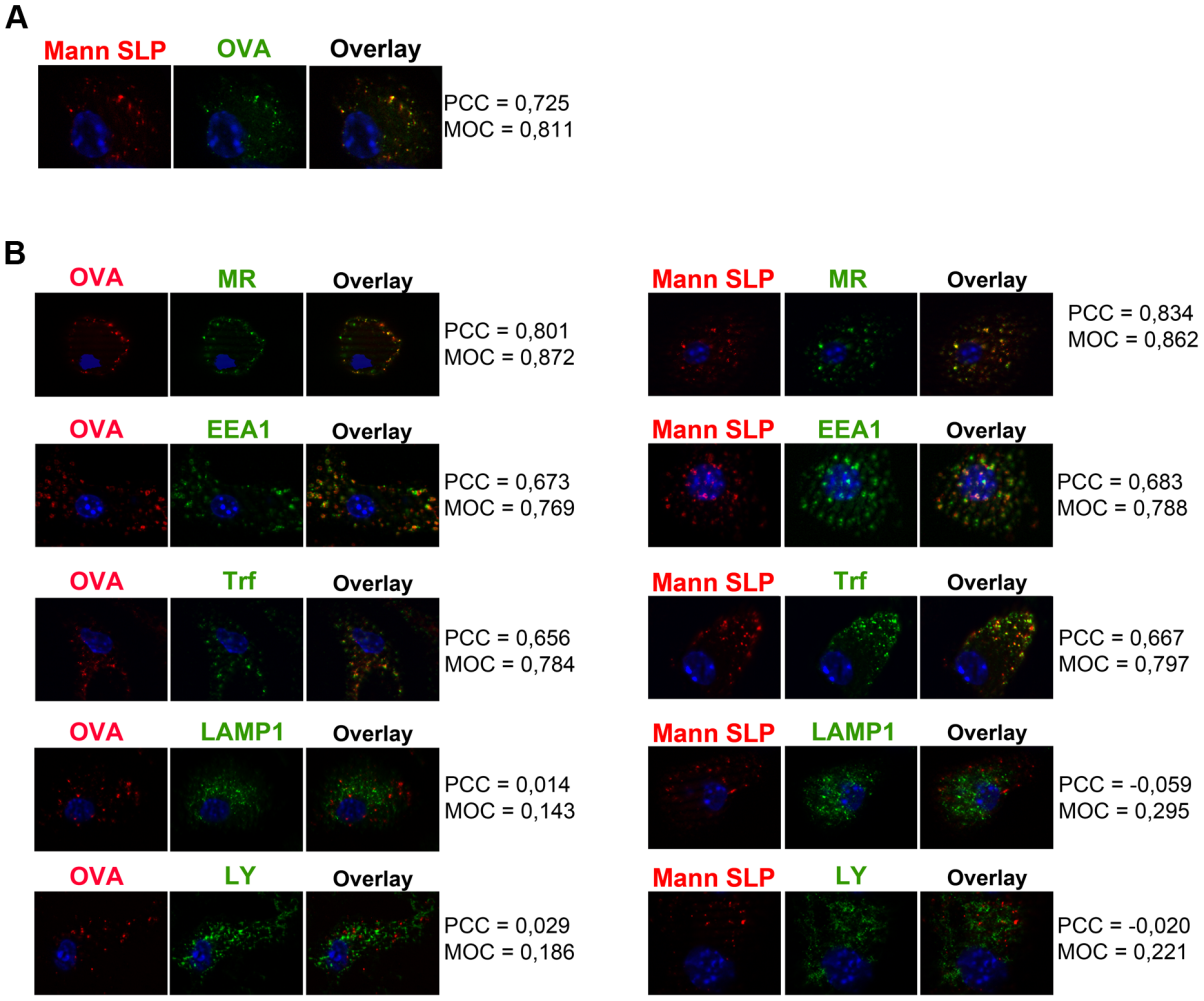
Intracellular localization of mannosylated SLPs. A) Wild-type BM-DCs were incubated simultaneously with Alexa647-labeled SLPs and Alexa488-labeled OVA for 15 min. After medium chase of another 20 min, intracellular localization was determined by immunofluorescence microscopy. B) Wild-type BM-DCs were incubated with fluorochrome-labeled OVA, SLPs, Transferrin and/or Lucifer Yellow for 15 min, chased with medium for another 20 min and stained with antibodies against EEA1, LAMP1 or Lucifer Yellow. Intracellular distribution was analyzed by immunofluorescence microscopy. To analyse co-localization of OVA and SLPs with the indicated markers, the Pearson correlation coefficient (varying between −1 and +1 with −1 for perfect negative correlation, 0 for perfect absence of correlation and 1 for perfect correlation) and the Mander's overlap coefficient (varying between 0 and 1 with 0 for no overlap and +1 for perfect overlap), were calculated. Nuclei stained with DAPI are depicted in blue. PCC: Pearson Correlation Coefficient. MOC: Mander's overlap coefficient.

Since the MR targets internalized antigens towards an early endosomal compartment, from where efficient cross-presentation takes place, we questioned whether mannosylation might target SLPs towards the same endocytic compartment. Therefore, we first incubated DCs simultaneously with fluorochrome-labeled SLPs and OVA, which in BM-DCs is targeted by the MR into these early endosomes [17], and analyzed their intracellular distribution by immunofluorescence microscopy. Figure 3A demonstrates a clear co-localization of mannosylated SLPs and OVA, indicating that mannosylation might indeed target SLPs towards early endosomes. To verify this hypothesis, we analyzed the co-localization of mannosylated peptides with different endosome markers. As for OVA, a clear co-localization of mannosylated SLPs with the MR itself and with the early endosome markers early endosome antigen 1 (EEA1) and transferrin (Trf) was observed, whereas the lysosomal marker Lysosomal-associated membrane protein 1 (LAMP1) and the pinocytosis marker lucifer yellow (LY), which upon internalization is rapidly targeted towards lysosomes, do not co-localize with mannosylated SLPs (Fig. 3B). These observations demonstrate that increased uptake of mannosylated SLPs by the MR indeed targets them towards early endosomes.

### Increased cross-presentation but not MHC II-restricted presentation of mannosylated SLPs

**Figure 4:**
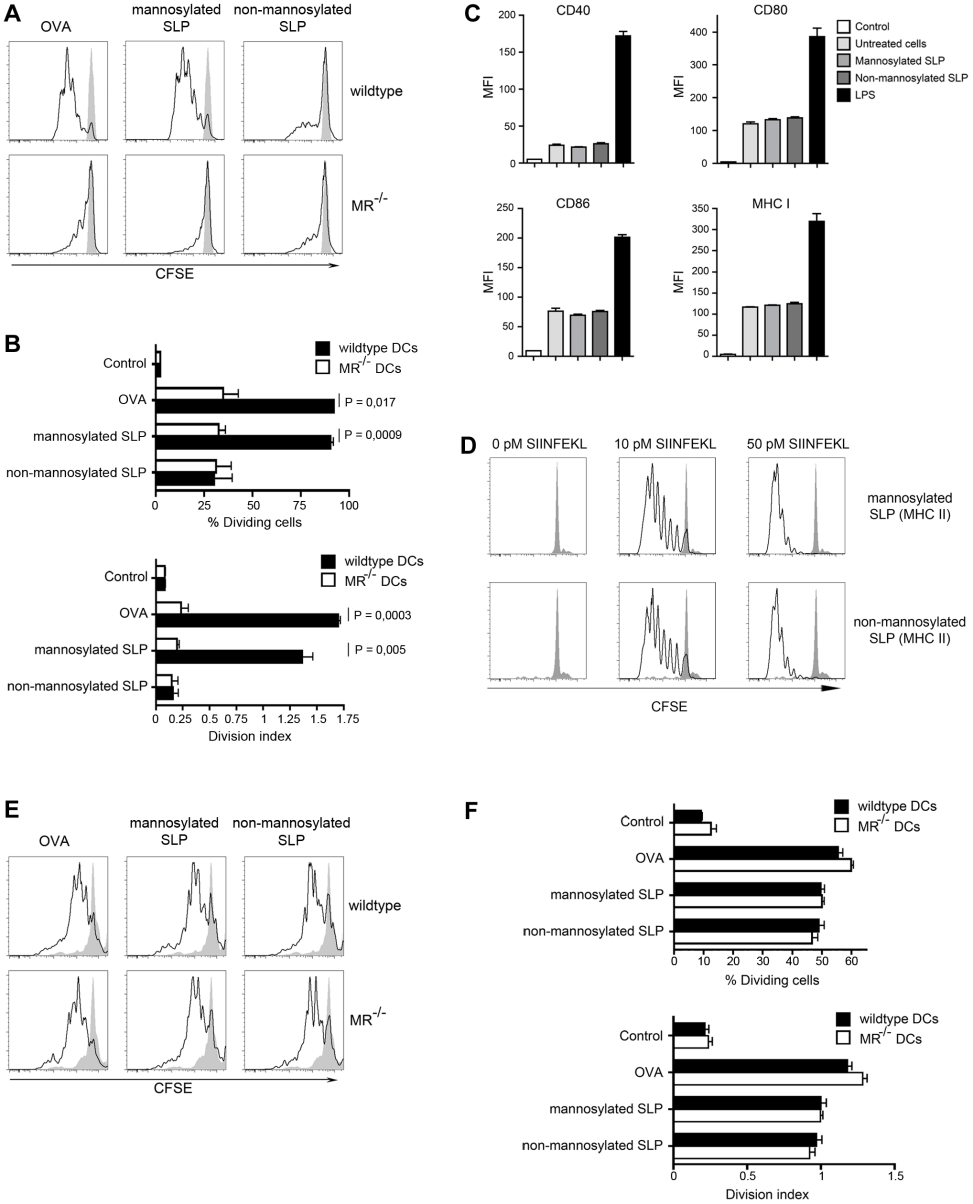
Cross-presentation and MHC II-restricted presentation of mannosylated SLPs. A) Wildtype or MR-deficient BM-DCs were incubated with OVA or SLPs and incubated with CFSE-labeled OT-I T cells. After 3 days, T cell proliferation was determined by flow cytometry after gating on CD8^+^ cells. B) Quantification of A), depicting the division index and the percentage of dividing OT-I T cells. C) BM-DCs were treated with 100 ng/ml LPS or 50 nM mannosylated or non-mannosylated SLPs. After 24 h, expression of CD40, CD80, CD86 and MHC I were analyzed by flow cytometry. Control: Isotype control. D) BM-DCs were pre-treated with 50 nM mannosylated or non-mannosylated SLPs containing the MHC II epitope of OVA for 1 h before they were loaded with the SIINFEKL short peptide for another 3 h and incubated with CFSE-labeled OT-I T cells. After 3 days, T cell proliferation was determined by flow cytometry after gating on CD8^+^ cells. E) Wildtype or MR-deficient BM-DCs were incubated with OVA or SLPs and incubated with CFSE-labeled OT-II T cells. After 3 days, T cell proliferation was determined by flow cytometry after gating on CD4^+^ cells. F) Quantification of E), depicting the division index and the percentage of dividing OT-II cells. Depicted are representative results of at least 3 independent experiments.

Since antigens targeted towards early endosomes by the MR are efficiently processed for cross-presentation [17], we examined whether increased MR-mediated endocytosis and targeting towards early endosomes might also enhance cross-presentation of mannosylation of SLPs. Therefore, we treated wild-type or MR-deficient DCs with mannosylated or non-mannosylated SLPs containing the MHC I-restricted epitope OVA_257–264_. Subsequently, we incubated the DCs with CFSE-labeled OVA-specific CD8^+^ T cells (OT-I) and monitored T cell proliferation by flow cytometry. In wild-type DCs, poor T cell proliferation was observed after DC treatment with non-mannosylated SLPs (Fig. 4A, quantification in Fig. 4B). After treatment with mannosylated SLPs, however, a strong T cell proliferation could be monitored. This proliferation was comparable to samples where DCs were treated with OVA and was not detected in the absence of the MR, demonstrating that MR-mediated endocytosis of mannosylated SLPs severely enhanced cross-presentation and concomitant CD8^+^ T cell activation.

To exclude that the observed increase in cross-presentation was due to an altered maturation of DCs after incubation with mannosylated SLPs, we first analyzed the up-regulation of co-stimulatory molecules and MHC I after treatment with mannosylated or non-mannosylated SLPs. In contrast to LPS, which induced a strong up-regulation of CD40, CD80, CD86 and MHC I, the addition of SLPs did not alter the expression of these molecules, regardless whether SLPs were mannosylated or not (Fig. 4C), pointing out that the addition of mannosylated SLPs did not alter DC maturation. To fully exclude any effects of mannosylated ligands on the overall capacities of DCs to activate T cells, we pre-treated DCs with mannosylated or non-mannosylated SLPs containing the MHC II-restricted epitope of OVA (peptides 3 and 4 in figure 1). Afterwards, we loaded these DCs with a non-mannosylated (short) peptide, containing only the MHC I-restricted epitope SIINFEKL, and analyzed proliferation of OT-I T cells. Importantly, no effect on OT-I T cell proliferation was observed after pre-treatment with mannosylated SLPs (Fig. 4D), demonstrating that the overall capacities of DCs to activate T cells is not influenced by the addition of mannosylated SLPs.

Next, we investigated the effect of SLP mannosylation on MHC II-restricted presentation. To this end, we incubated wildtype or MR-deficient DCs with mannosylated or non-mannosylated SLPs containing the MHC II-restricted epitope OVA_323–339_. Subsequent incubation with CFSE-labeled OVA-specific CD4^+^ T cells (OT-II) revealed that mannosylation of SLPs did not influence MHC II-restricted presentation (Fig. 4E, quantification in Fig. 4F). Consistently, the presence of the MR did not affect MHC II-restricted presentation of (mannosylated or non-mannosylated) SLPs, demonstrating that the enhancing effect of mannosylation is restricted to antigen cross-presentation and confirming again that the overall capacities of DCs to activate T cells remain unaltered by the addition of mannosylated SLPs.

### Increased proliferation of CD8^+^ T cells in vivo after treatment with mannosylated SLPs

**Figure 5:**
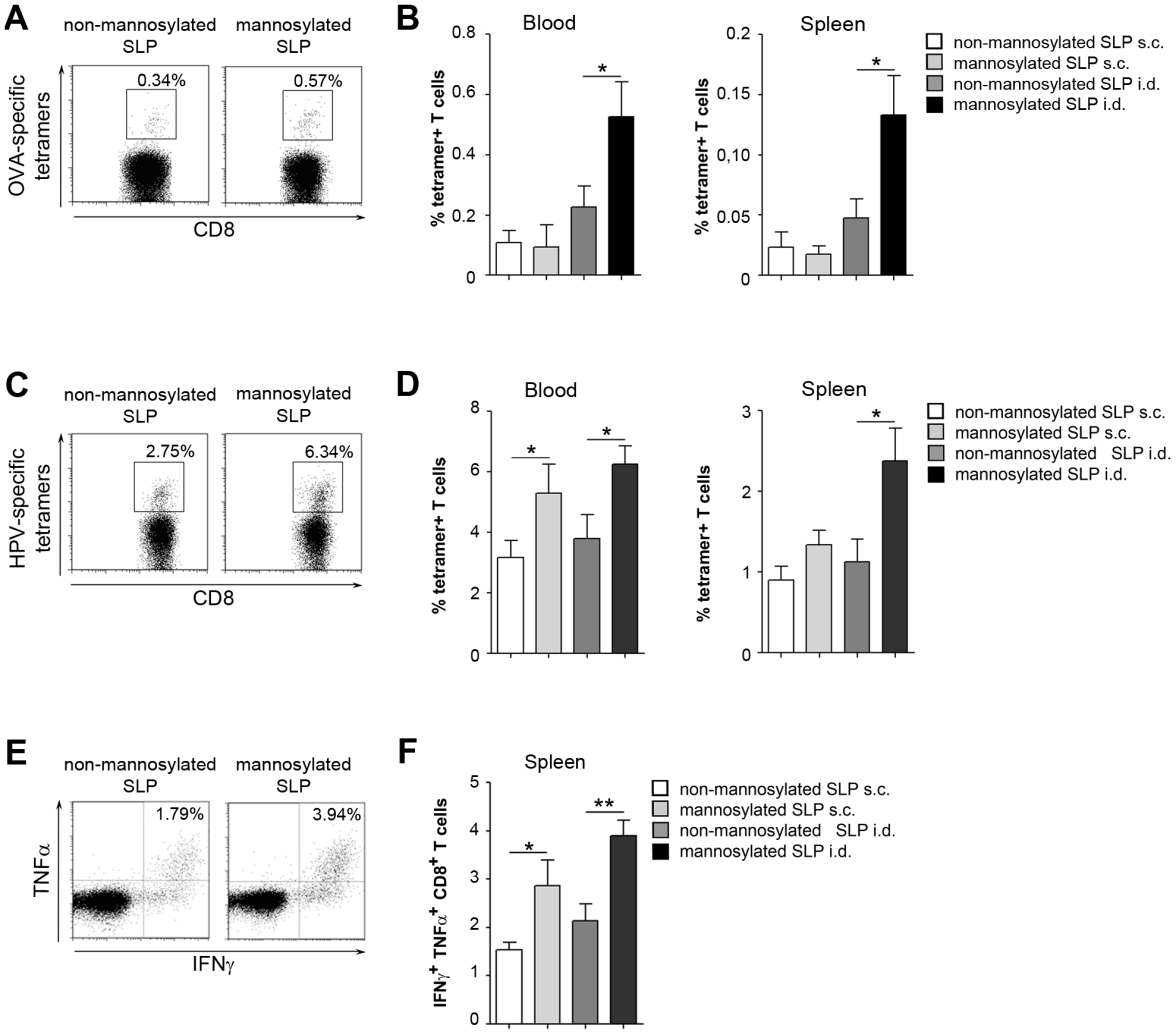
In vivo T cell activation by mannosylated SLPs. A) Mice were injected i.d. with 75 µg of OVA-specific mannosylated or non-mannosylated SLPs and 20 µg CpG. After 7 days, antigen-specific T cells in the blood were monitored by flow cytometry using epitope-specific tetramers. Cells were gated for CD8. B) Quantitative analysis of epitope-specific T cells in the blood or in the spleen after s.c or i.d. injection of SLPs. Graphs show percentage of tetramer-positive CD8^+^ T cells. C) as in A) using HPV-specific SLPs. D) Quantitative analysis of epitope-specific T cells in the blood or in the spleen after s.c or i.d. injection of HPV-specific SLPs. E) Intracellular cytokine staining of splenic CD8^+^ T cells after i.d. injection of HPV-specific mannosylated or non-mannosylated peptides and CpG as above. F) Quantitative analysis of intracellular cytokines in T cells isolated from the spleen after s.c or i.d. injection of HPV-specific SLPs. Graphs show percentage of IFNγ^+^ TNFα^+^ cells amongst all CD8^+^ T cells. Dot plots depict representative results of 2 independent experiments. Bar graphs depict pooled results of 2 independent experiments (n = 9–10).

Finally, we aimed at investigating the effect of mannosylating SLPs on cross-presentation *in vivo*. Therefore, we first injected mice subcutaneously (s.c.) or intradermally (i.d.) with mannosylated or non-mannosylated SLPs containing the MHC I-restricted OVA epitope (peptides 1 and 2 in Fig. 1) and used CpG as an adjuvant. After 7 days, we used MHC class I tetramer staining to monitor the frequency of antigen-specific T cells in the blood and spleen. Whereas no differences in T cell responses could be observed between s.c. injection of mannosylated versus non-mannosylated SLPs, i.d. injection of mannosylated SLPs induced pronounced CD8^+^ T cell responses compared to non-mannosylated SLPs in both the blood and the spleen (Fig. 5A and 5B). These results point out that mannosylation of SLPs indeed can enhance cross-presentation of SLPs, especially if they are injected intradermally.

To investigate whether these differences also hold true for other antigens, we used mannosylated and non-mannosylated SLPs containing a HVP16 E7 epitope. As for SLPs containing OVA epitopes, we injected these peptides s.c. or i.d. into recipient mice and monitored CD8^+^ T cell expansion. Consistently to the results obtained for the SLPs containing the OVA epitope, mannosylated SLPs bearing the HPV epitope elucidated an enhanced antigen-specific T cell expansion compared to non-mannosylated SLPs in both blood and spleen (Fig. 5C and 5D). These differences were most pronounced if the SLPs were i.d. injected. Differences in T cell numbers were also observed after immunization in the absence of CpG, demonstrating that they were not due to a differential effect of CpG (Fig. S2).

Additional to an increased expansion, T cells activated after injection of mannosylated SLPs depicted an increased expression of IFN-γ and TNFα compared to non-mannosylated SLPs (Fig. 5E and 5F), pointing out an increased T cell activation after injection with mannosylated SLPs.

## Discussion

In order to be cross-presented efficiently, SLPs need to be internalized and processed within the DC [22]. Since the stability of loaded MHC I molecules at the cell membrane is limited [23] but migration of the DCs towards the draining lymph nodes for T cell activation is a time-consuming process, it is essential that continuous cross-presentation occurs to supply the DC with freshly loaded MHC I molecules. Such persistent cross-presentation depends on prolonged antigen stability, since prompt antigen degradation by endo/lysosomal proteases rapidly destroys putative MHC I epitopes [24], [25]. Therefore, it has been proposed that for efficient cross-presentation, antigens are targeted towards specific antigen storage compartments, where they are protected from rapid degradation by lysosomal proteases and from where continuous processing can take place [25]. Since the MR specifically targets its ligands towards a non-degradative subset of early endosomes [17], [26], [27], from where processing for cross-presentation efficiently can take place [18], [19], the MR seems to be an optimal target for vaccination purposes aimed at the induction of a cytotoxic CD8^+^ T cell response [28], [29].

In this study, we demonstrated that mannosylation of SLPs increased their MR-mediated uptake, resulting in enhanced cross-presentation and CD8^+^ T cell activation both *in vitro* and *in vivo*. Increased cross-presentation was not due to an altered overall capacity of the DC to activate T cells caused by mannosylated ligands, as the expression of co-stimulatory molecules remained unaffected and the addition of an irrelevant mannosylated SLP did not alter proliferation of OT-I cells. Interestingly, increased CD8^+^ T cell activation was most pronounced after injecting the SLPs i.d., whereas s.c. injection of mannosylated SLPs only lead to a marginal difference compared to non-mannosylated SLPs. These differences most likely can be explained by a differential MR expression in the differential parts of the skin. Whereas dermal DCs express high levels of MR [20], no MR expression is observed in Langerhans cells of the epidermis [30].

In accordance with increased cross-presentation and activation of cytotoxic T cells by mannosylated SLPs observed here, targeting of the breast cancer mucin MUC1 towards the MR resulted in enhanced cross-presentation, an increased CD8^+^ T cell cytotoxicity and a reduced humoral response.

In a previous study, we demonstrated that in murine BM-DCs low amounts of antigens internalized by fluid phase pinocytosis were targeted rapidly towards lysosomes, where they co-localized with and were presented very efficiently on MHC II molecules [17]. In contrast to pinocytosed antigens, MR-internalized antigens were not targeted towards MHC II^+^ compartments and did not contribute to MHC II-restricted antigen presentation [17]. Similarly, in this study, low amounts of non-mannosylated SLPs were presented very efficiently on MHC II, whereas increased uptake after mannosylation specifically enhanced MR-mediated cross-presentation without influencing MHC II-restricted presentation, confirming the close correlation of the MR with cross-presentation. This correlation might also explain the limited success of mannosylated antigens in animal models like experimental autoimmune encephalomyelitis (EAE) [31], [32], [33], which is known to be mediated mainly by CD4^+^ T cells. Nevertheless, some studies also report of increased MHC II-restricted antigen presentation of MR-internalized antigens [34], [35]. In these studies, the authors conjugated their antigens to MR-specific antibodies. However, cross-linking of the MR by the use of MR-specific antibodies influences DC maturation [36] and might also alter antigen routing and presentation as shown for dectin-1, another c-type lectin receptor. Whereas monovalent dectin-1 ligands are targeted towards early endosomes for cross-presentation, polyvalent ligands are rapidly targeted towards lysosomes, resulting in increased MHC II-restricted presentation [37]. These findings point out that receptor cross-linking regulates intracellular routing and presentation of internalized antigens and might be an alternative explanation for the observed MHC II-restricted presentation resulting from antibody-mediated MR-targeting. Similarly, increased MHC II-restricted presentation of mannosylated peptides or mannosylated BSA by human monocyte-derived DCs has been observed [38], [39]. These cells, however, express additional C-type lectin receptors like DC-SIGN [40], which is not expressed on murine BM-DCs. Since DC-SIGN also binds and internalizes mannosylated ligands [40], [41] and DC-SIGN-mediated endocytosis leads to efficient presentation on MHC II [40], it is feasible that MHC II presentation observed in these studies has been the result of antigen internalization via DC-SIGN.

Taken together, we could demonstrate that mannosylation of SLPs causes increased MR-mediated uptake and antigen routing into early endosomes, resulting in efficient cross-presentation and enhanced CD8^+^ T cell activation *in vivo* and *in vitro*. These findings might help to optimize vaccination strategies aimed at the induction of a cytotoxic T cell response in e.g. anti-tumor and anti-viral therapies.

## Supporting Information

Figure S1 **Uptake of mannosylated or non-mannosylated SLP in time course experiment.** Wildtype or MR-deficient BM-DCs were incubated with 200 nM mannosylated SLPs or non-mannosylated SLPs for the indicated time points. Antigen uptake was monitored by flow cytometry (gated on all living cells).
(DOCX)

Figure S2 **Immunization with mannosylated or non-mannosylated SLP in the absence of CpG.** Mice were injected i.d. with 75 µg of HPV-specific mannosylated or non-mannosylated SLPs in the absence of CpG. After 7 days, antigen-specific T cells in the blood were monitored by flow cytometry using epitope-specific tetramers. Bars show percentage of tetramer^+^ T cells.
(DOCX)

## References

[1] De Gregorio E, Rappuoli R (2012) Vaccines for the future: learning from human immunology. Microb Biotechnol 5: 149–155. 21880117 10.1111/j.1751-7915.2011.00276.x PMC3815775

[2] Arens R (2012) Rational design of vaccines: learning from immune evasion mechanisms of persistent viruses and tumors. Adv Immunol 114: 217–243. 22449784 10.1016/B978-0-12-396548-6.00009-3

[3] Arens R, van Hall T, van der Burg SH, Ossendorp F, Melief CJ (2013) Prospects of combinatorial synthetic peptide vaccine-based immunotherapy against cancer. Seminars in immunology 25: 182–190. 23706598 10.1016/j.smim.2013.04.008

[4] Melief CJ, van der Burg SH (2008) Immunotherapy of established (pre)malignant disease by synthetic long peptide vaccines. Nat Rev Cancer 8: 351–360. 18418403 10.1038/nrc2373

[5] van Hall T, van der Burg SH (2012) Mechanisms of peptide vaccination in mouse models: tolerance, immunity, and hyperreactivity. Adv Immunol 114: 51–76. 22449778 10.1016/B978-0-12-396548-6.00003-2

[6] Jung S, Unutmaz D, Wong P, Sano G, De los Santos K, et al (2002) In vivo depletion of CD11c+ dendritic cells abrogates priming of CD8+ T cells by exogenous cell-associated antigens. Immunity 17: 211–220. 12196292 10.1016/s1074-7613(02)00365-5 PMC3689299

[7] Toes RE, Offringa R, Blom RJ, Melief CJ, Kast WM (1996) Peptide vaccination can lead to enhanced tumor growth through specific T-cell tolerance induction. Proceedings of the National Academy of Sciences of the United States of America 93: 7855–7860. 8755566 10.1073/pnas.93.15.7855 PMC38838

[8] Zwaveling S, Ferreira Mota SC, Nouta J, Johnson M, Lipford GB, et al (2002) Established human papillomavirus type 16-expressing tumors are effectively eradicated following vaccination with long peptides. Journal of immunology 169: 350–358. 10.4049/jimmunol.169.1.350 12077264

[9] Bijker MS, van den Eeden SJ, Franken KL, Melief CJ, Offringa R, et al (2007) CD8+ CTL priming by exact peptide epitopes in incomplete Freund's adjuvant induces a vanishing CTL response, whereas long peptides induce sustained CTL reactivity. Journal of immunology 179: 5033–5040. 10.4049/jimmunol.179.8.5033 17911588

[10] Kenter GG, Welters MJ, Valentijn AR, Lowik MJ, Berends-van der Meer DM, et al (2009) Vaccination against HPV-16 oncoproteins for vulvar intraepithelial neoplasia. N Engl J Med 361: 1838–1847. 19890126 10.1056/NEJMoa0810097

[11] Smith CM, Bradding P, Neill DR, Baxendale H, Felici F, et al (2011) Novel immunogenic peptides elicit systemic anaphylaxis in mice: implications for peptide vaccines. Journal of immunology 187: 1201–1206. 10.4049/jimmunol.1002152 21709154

[12] Welters MJ, Bijker MS, van den Eeden SJ, Franken KL, Melief CJ, et al (2007) Multiple CD4 and CD8 T-cell activation parameters predict vaccine efficacy in vivo mediated by individual DC-activating agonists. Vaccine 25: 1379–1389. 17123670 10.1016/j.vaccine.2006.10.049

[13] Zom GG, Khan S, Filippov DV, Ossendorp F (2012) TLR ligand-peptide conjugate vaccines: toward clinical application. Adv Immunol 114: 177–201. 22449782 10.1016/B978-0-12-396548-6.00007-X

[14] van Duikeren S, Fransen MF, Redeker A, Wieles B, Platenburg G, et al (2012) Vaccine-induced effector-memory CD8+ T cell responses predict therapeutic efficacy against tumors. Journal of immunology 189: 3397–3403. 10.4049/jimmunol.1201540 22914049

[15] Burgdorf S, Lukacs-Kornek V, Kurts C (2006) The mannose receptor mediates uptake of soluble but not of cell-associated antigen for cross-presentation. J Immunol 176: 6770–6776. 16709836 10.4049/jimmunol.176.11.6770

[16] Ezekowitz RA, Sastry K, Bailly P, Warner A (1990) Molecular characterization of the human macrophage mannose receptor: demonstration of multiple carbohydrate recognition-like domains and phagocytosis of yeasts in Cos-1 cells. J Exp Med 172: 1785–1794. 2258707 10.1084/jem.172.6.1785 PMC2188777

[17] Burgdorf S, Kautz A, Bohnert V, Knolle PA, Kurts C (2007) Distinct pathways of antigen uptake and intracellular routing in CD4 and CD8 T cell activation. Science 316: 612–616. 17463291 10.1126/science.1137971

[18] Burgdorf S, Scholz C, Kautz A, Tampe R, Kurts C (2008) Spatial and mechanistic separation of cross-presentation and endogenous antigen presentation. Nature immunology 9: 558–566. 18376402 10.1038/ni.1601

[19] Zehner M, Chasan AI, Schuette V, Embgenbroich M, Quast T, et al (2011) Mannose receptor polyubiquitination regulates endosomal recruitment of p97 and cytosolic antigen translocation for cross-presentation. Proceedings of the National Academy of Sciences of the United States of America 108: 9933–9938. 21628571 10.1073/pnas.1102397108 PMC3116392

[20] Keler T, Ramakrishna V, Fanger MW (2004) Mannose receptor-targeted vaccines. Expert Opin Biol Ther 4: 1953–1962. 15571457 10.1517/14712598.4.12.1953

[21] Harvey DJ, Wing DR, Kuster B, Wilson IB (2000) Composition of N-linked carbohydrates from ovalbumin and co-purified glycoproteins. J Am Soc Mass Spectrom 11: 564–571. 10833030 10.1016/S1044-0305(00)00122-7

[22] Faure F, Mantegazza A, Sadaka C, Sedlik C, Jotereau F, et al (2009) Long-lasting cross-presentation of tumor antigen in human DC. European journal of immunology 39: 380–390. 19130478 10.1002/eji.200838669

[23] van Montfoort N, Camps MG, Khan S, Filippov DV, Weterings JJ, et al (2009) Antigen storage compartments in mature dendritic cells facilitate prolonged cytotoxic T lymphocyte cross-priming capacity. Proceedings of the National Academy of Sciences of the United States of America 106: 6730–6735. 19346487 10.1073/pnas.0900969106 PMC2672553

[24] Savina A, Jancic C, Hugues S, Guermonprez P, Vargas P, et al (2006) NOX2 controls phagosomal pH to regulate antigen processing during crosspresentation by dendritic cells. Cell 126: 205–218. 16839887 10.1016/j.cell.2006.05.035

[25] Cebrian I, Visentin G, Blanchard N, Jouve M, Bobard A, et al (2011) Sec22b regulates phagosomal maturation and antigen crosspresentation by dendritic cells. Cell 147: 1355–1368. 22153078 10.1016/j.cell.2011.11.021

[26] Wainszelbaum MJ, Proctor BM, Pontow SE, Stahl PD, Barbieri MA (2006) IL4/PGE2 induction of an enlarged early endosomal compartment in mouse macrophages is Rab5-dependent. Experimental cell research 312: 2238–2251. 16650848 10.1016/j.yexcr.2006.03.025

[27] Chatterjee B, Smed-Sorensen A, Cohn L, Chalouni C, Vandlen R, et al (2012) Internalization and endosomal degradation of receptor-bound antigens regulate the efficiency of cross presentation by human dendritic cells. Blood 120: 2011–2020. 22791285 10.1182/blood-2012-01-402370

[28] Apostolopoulos V, Pietersz GA, Gordon S, Martinez-Pomares L, McKenzie IF (2000) Aldehyde-mannan antigen complexes target the MHC class I antigen-presentation pathway. European journal of immunology 30: 1714–1723. 10898509 10.1002/1521-4141(200006)30:6<1714::AID-IMMU1714>3.0.CO;2-C

[29] Apostolopoulos V, Pietersz GA, McKenzie IF (1996) Cell-mediated immune responses to MUC1 fusion protein coupled to mannan. Vaccine 14: 930–938. 8843637 10.1016/0264-410x(95)00258-3

[30] Mommaas AM, Mulder AA, Jordens R, Out C, Tan MC, et al (1999) Human epidermal Langerhans cells lack functional mannose receptors and a fully developed endosomal/lysosomal compartment for loading of HLA class II molecules. European journal of immunology 29: 571–580. 10064073 10.1002/(SICI)1521-4141(199902)29:02<571::AID-IMMU571>3.0.CO;2-E

[31] Kel JM, de Geus ED, van Stipdonk MJ, Drijfhout JW, Koning F, et al (2008) Immunization with mannosylated peptide induces poor T cell effector functions despite enhanced antigen presentation. International immunology 20: 117–127. 18024466 10.1093/intimm/dxm123

[32] Kel J, Oldenampsen J, Luca M, Drijfhout JW, Koning F, et al (2007) Soluble mannosylated myelin peptide inhibits the encephalitogenicity of autoreactive T cells during experimental autoimmune encephalomyelitis. Am J Pathol 170: 272–280. 17200200 10.2353/ajpath.2007.060335 PMC1762692

[33] Luca ME, Kel JM, van Rijs W, Wouter Drijfhout J, Koning F, et al (2005) Mannosylated PLP(139–151) induces peptide-specific tolerance to experimental autoimmune encephalomyelitis. Journal of neuroimmunology 160: 178–187. 15710471 10.1016/j.jneuroim.2004.11.014

[34] He LZ, Crocker A, Lee J, Mendoza-Ramirez J, Wang XT, et al (2007) Antigenic targeting of the human mannose receptor induces tumor immunity. Journal of immunology 178: 6259–6267. 10.4049/jimmunol.178.10.6259 17475854

[35] McKenzie EJ, Taylor PR, Stillion RJ, Lucas AD, Harris J, et al (2007) Mannose receptor expression and function define a new population of murine dendritic cells. Journal of immunology (Baltimore, Md 178: 4975–4983. 10.4049/jimmunol.178.8.4975 17404279

[36] Chieppa M, Bianchi G, Doni A, Del Prete A, Sironi M, et al (2003) Cross-linking of the mannose receptor on monocyte-derived dendritic cells activates an anti-inflammatory immunosuppressive program. Journal of immunology 171: 4552–4560. 10.4049/jimmunol.171.9.4552 14568928

[37] Herre J, Marshall AS, Caron E, Edwards AD, Williams DL, et al (2004) Dectin-1 uses novel mechanisms for yeast phagocytosis in macrophages. Blood 104: 4038–4045. 15304394 10.1182/blood-2004-03-1140

[38] Tan MC, Mommaas AM, Drijfhout JW, Jordens R, Onderwater JJ, et al (1997) Mannose receptor-mediated uptake of antigens strongly enhances HLA class II-restricted antigen presentation by cultured dendritic cells. Eur J Immunol 27: 2426–2435. 9341789 10.1002/eji.1830270942

[39] Engering AJ, Cella M, Fluitsma D, Brockhaus M, Hoefsmit EC, et al (1997) The mannose receptor functions as a high capacity and broad specificity antigen receptor in human dendritic cells. Eur J Immunol 27: 2417–2425. 9341788 10.1002/eji.1830270941

[40] Engering A, Geijtenbeek TB, van Vliet SJ, Wijers M, van Liempt E, et al (2002) The dendritic cell-specific adhesion receptor DC-SIGN internalizes antigen for presentation to T cells. J Immunol 168: 2118–2126. 11859097 10.4049/jimmunol.168.5.2118

[41] Mitchell DA, Fadden AJ, Drickamer K (2001) A novel mechanism of carbohydrate recognition by the C-type lectins DC-SIGN and DC-SIGNR. Subunit organization and binding to multivalent ligands. The Journal of biological chemistry 276: 28939–28945. 11384997 10.1074/jbc.M104565200

