# Supplementary material for: Enhanced Cross-Presentation and Improved CD8+ T Cell Responses after Mannosylation of Synthetic Long Peptides in Mice
Source: PLoS One. 2014 Aug 19;9(8):e103755. doi: 10.1371/journal.pone.0103755 (PMC4138033; doi:10.1371/journal.pone.0103755)
Supplement: Figure S1 — Uptake of mannosylated or non-mannosylated SLP in time course experiment. Wildtype or MR-deficient BM-DCs were incubated with 200 nM mannosylated SLPs or non-mannosylated SLPs for the indicated time points. Antigen uptake was monitored by flow cytometry (gated on all living cells). (DOCX) [file pone.0103755.s001.docx]

**Figure S1**


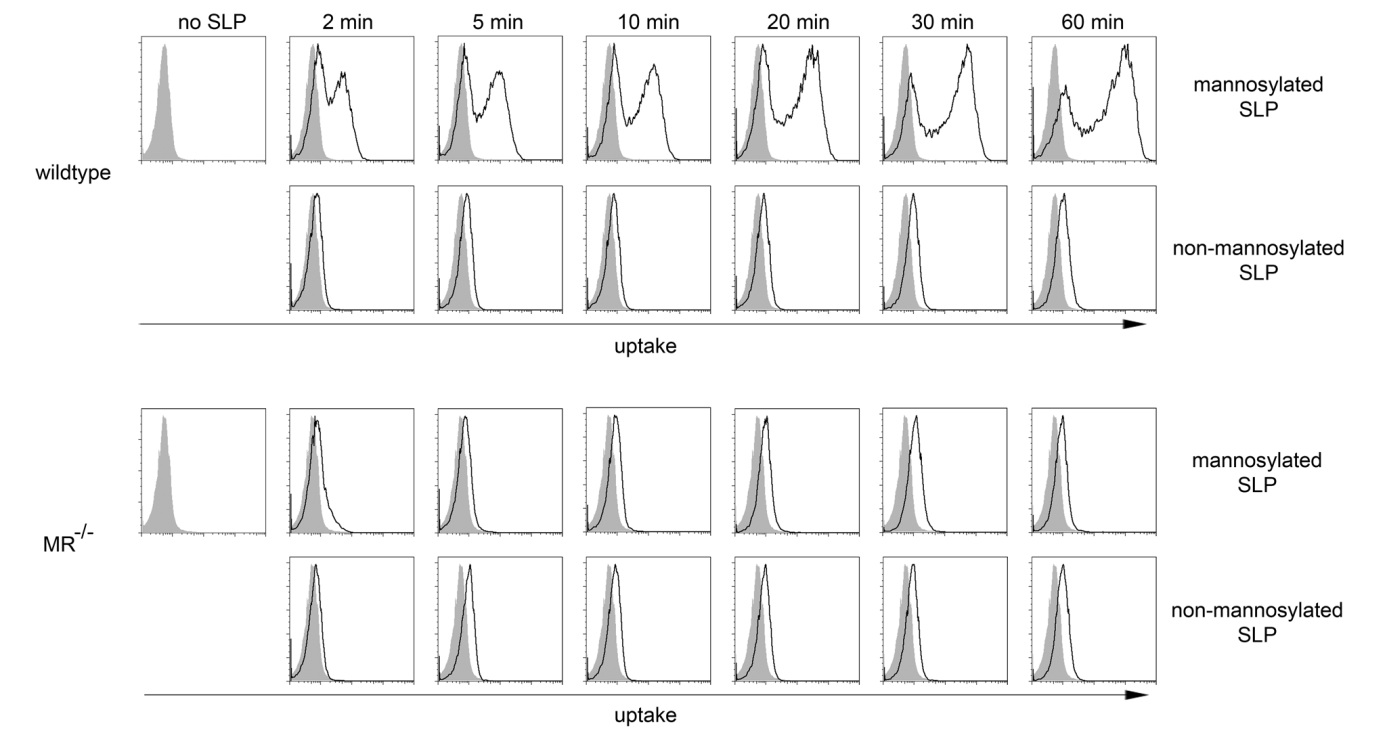


***Figure S1: Uptake of mannosylated or non-mannosylated SLP in time course experiment***

Wildtype or MR-deficient BM-DCs were incubated with 200 nM mannosylated SLPs or non-mannosylated SLPs for the indicated time points. Antigen uptake was monitored by flow cytometry (gated on all living cells).
