# Supplementary material for: Enhanced Cross-Presentation and Improved CD8+ T Cell Responses after Mannosylation of Synthetic Long Peptides in Mice
Source: PLoS One. 2014 Aug 19;9(8):e103755. doi: 10.1371/journal.pone.0103755 (PMC4138033; doi:10.1371/journal.pone.0103755)
Supplement: Figure S2 — Immunization with mannosylated or non-mannosylated SLP in the absence of CpG. Mice were injected i.d. with 75 µg of HPV-specific mannosylated or non-mannosylated SLPs in the absence of CpG. After 7 days, antigen-specific T cells in the blood were monitored by flow cytometry using epitope-specific tetramers. Bars show percentage of tetramer+ T cells. (DOCX) [file pone.0103755.s002.docx]

**Figure S2**


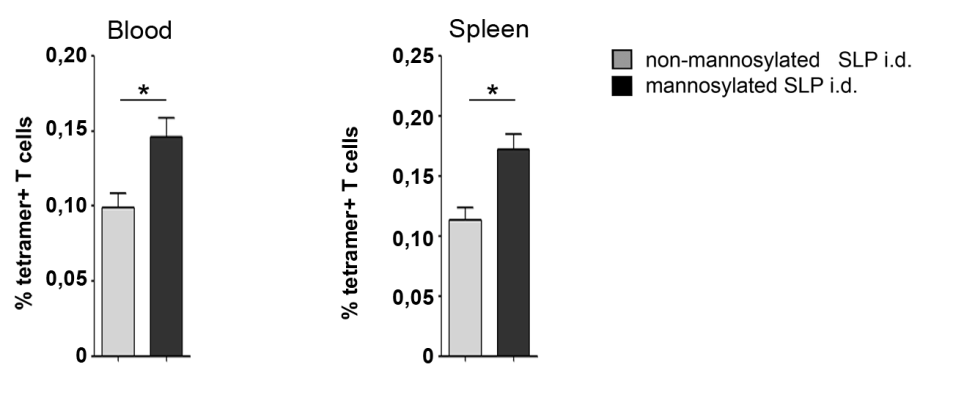


***Figure S2: Immunization with mannosylated or non-mannosylated SLP in the absence of CpG***

Mice were injected i.d. with 75 μg of HPV-specific mannosylated or non-mannosylated SLPs in the absence of CpG. After 7 days, antigen-specific T cells in the blood were monitored by flow cytometry using epitope-specific tetramers. Bars show percentage of tetramer^+^ T cells.
